# Lympho-Sarcoma

**Published:** 1890-04

**Authors:** S. D. Brimhall

**Affiliations:** Minneapolis, Minn.


### LYMPHO-SARCOMA.

By S. D. Brimhall, V. M. D.,

Minneapolis, Minn.

Subject: A bay mare 9 years old, weighing about 900 pounds.

*History.*—Had not been driving as free as usual for a month or more, then began having a diarrhœa which did not improve, although given change of food and rest for three weeks or more. At this time I found her in fair flesh; membranes healthy; pulse, respiration and temperature normal; appetite capricious and great thirst; fluid fæces without bad odor were frequently passed. Her teeth being irregular were floated, and gave astringents in full doses.

In about a week I saw her again, but found no improvement. I learned that after drinking a half pail of water (that being all she was allowed at a time) her bowels would become very active in their movements, and in half an hour there would be a free discharge of liquid fæces. By decreasing the allowance of water and giving very powerful astringents for twenty-four hours the fæces became normal in form, *i. e.*, in balls, but their smell was very offensive. There was also a rise of temperature to 104½°, pulse 70, weak and small; respiration a little increased. I continued the medicine twenty-four hours more, and increased the allowance of water, which was very soon followed by liquid fæces; and temperature, etc., gradually dropped to normal. I was now quite sure the diarrhœa was due to some irritating body in the intestines, and by a rectal examination could just reach a large irregular mass (which could not be broken with the fingers) situated in the right hypochondriac region, located, I thought, in the cæcum.

The owner, not wishing to kill her, sent her to the country, although she was getting weak and failing in flesh fast, as her appetite was very poor, and what she did eat did her little good. In a few days the owner asked me to go out and kill the mare, which I did, and on examination found a large tumor on the inside of cæcum opposite the ilio-cæcal valve, and filling this part of the cæcum, except a small space for passage of food from small intestine past the tumor into large colon. The lower part of cæcum contained a little fluid and the point had become gangrenous, with peritonitis of abdominal wall and diaphragm in contact with it. Along the course of the bloodvessels and lymphatics of cæcum and large colon there was a ridge as thick as a man's wrist, and at one point on the colon it had increased to the size of two fists. The liver also contained two small tumors.

Dr. J. C. Stewart, Professor of Histology at University of Minnesota, examined these tumors for me and found them to be lympho-sarcomas.

